# Genomic surveillance of the Chikungunya Virus (CHIKV) in Northeast Brazil after the first outbreak in 2014

**DOI:** 10.1590/0037-8682-0583-2019

**Published:** 2020-06-22

**Authors:** Ayslany Melo Rodrigues, Rafael Ribeiro Mota Souza, Larissa Moraes dos Santos Fonseca, Carolina de Araújo Rolo, Rejane Hughes Carvalho, Silvia Ines Sardi, Gubio Soares Campos

**Affiliations:** 1 Universidade Federal da Bahia, Laboratório de Virologia, Instituto de Ciências da Saúde, Salvador, BA, Brasil.; 2 Universidade Federal da Bahia, Laboratório de Virologia, Departamento de Biointeração, Instituto de Ciências da Saúde, Salvador, BA, Brasil.

**Keywords:** CHIKV, Phylogenetic analysis, Viral isolation

## Abstract

**INTRODUCTION:**

We performed an epidemiological surveillance of the Chikungunya (CHIKV) lineages in Bahia after the 2014 East/Central/South African (ECSA) genotype outbreak.

**METHODS:**

Reverse-transcription polymerase chain reaction (RT-PCR), viral isolation, and phylogenetic analyses were conducted on serum samples from 605 patients with CHIKV-like symptoms during 2014-2018.

**RESULTS:**

Of the 605 samples, 167 were CHIKV-positive. Viral isolation was achieved for 20 samples; their phylogenetic analysis (E2 protein) revealed the presence of ECSA lineage and reinforced the phylogenetic relationship between ECSA and Indian Ocean lineages.

**CONCLUSIONS:**

The genomic surveillance of CHIKV showed that only ECSA lineage circulated in Bahia since the 2014 outbreak.

Chikungunya virus (CHIKV) is a single-stranded positive-sense RNA virus belonging to the genus *Alphavirus* of the *Togaviridae* family. It is predominantly transmitted by *Aedes aegypti* and *Ae. albopictus* mosquitoes. The genome of this virus encodes four non-structural proteins (NSP 1-4) and three structural proteins (C, E1, and E2). Four CHIKV lineages have been identified, namely, the West African, East/Central/South African (ECSA), Asian (AL), and Indian Ocean (IOL) lineages^1^. CHIKV is an endemic arbovirus detected in 51 countries in the Americas. The clinical manifestations include high fever, rash, myalgia, and episodes of arthralgia, consequently leading to chronic pain and disability^1^. In Brazil, two autochthonous CHIKV cases were reported in September 2014, one caused by AL in Oiapoque, State of Amapa, North region, and another caused by ECSA in Feira de Santana, State of Bahia, Northeast region. Bahia was the first state in Brazil, and in general in the Americas, to detect the ECSA linage^2^. The ECSA lineage was probably introduced in Bahia through a traveler who returned from Angola, West Africa, to visit his family in Feira de Santana^2^. Bahia was also the first state in Brazil to identify and confirm the outbreak of the Zika virus (ZIKV) in 2015 that causes microcephaly abnormalities^3^. 

Since the CHIKV outbreak in 2014, the number of cases in Brazil has increased. The rapid spread of CHIKV and its co-circulation with other endemic arboviruses such as the Dengue virus and ZIKV may pose a serious public health problem in Brazil. Dual arboviral infections have been detected in patients residing in these endemic regions^4^. Furthermore, recent studies in Salvador, Bahia, have shown that CHIKV is not only associated with a severe chronic joint pain but also with the occurrence of encephalitis in children^5^.

The surveillance of the possible distinct CHIKV lineages in Brazil is imperative, particularly in Bahia, the epicenter of the ECSA outbreak in Brazil. In the present study, we report the outbreaks of CHIKV and describe the molecular genotype surveillance from 2014 to 2018 after the first episode in Bahia. 

In this study, serum samples were obtained from 605 patients (adults, male or female) with acute CHIKV-like symptoms (fever, rash, arthralgia, and headache) from public health care units in the city of Salvador and nearest locations (Camaçari, Feira de Santana, Coração de Maria) in Bahia. The samples were subjected to the molecular diagnosis of CHIKV at the Laboratory of Virology-Federal, University of Bahia. The study protocol was approved by the Comissão Nacional de Ética em Pesquisa em Brazil [the National Ethics Committee in Research, Brazil] (protocol CAEE 45483115.9.0000.0046; CAEE 82340017.9.0000.5662). 

Viral RNA was extracted from each sample using the QIAamp® Viral RNA Mini Kit and used for conventional reverse-transcription polymerase chain reaction (RT-PCR) on an Access RT-PCR System (Promega Co, Brazil) using a pair of E2 primers, as per a method described by Edwards et al^6^. A fragment of the *E2* gene (genomic position: 9403-9712; 305 bp) was amplified and visualized on a 2% agarose gel after staining with ethidium bromide and exposure to ultraviolet light. The CHIKV-positive serum samples (n = 20) in RT-PCR were used for viral isolation in the C6/36 culture cells (the Leibovitz’s L15 medium [Gibco®, Brazil] supplemented with 5% fetal bovine serum [Gibco®, Brazil]). CHIKV-positive sera (25 µL/mL) were inoculated in tubes with the C6/36 cells, and the cultures were maintained at 28°C for at least 7 days. The supernatant was collected, and the infection step was repeated thrice. After the third passage, the C6/36-infected cells were frozen, thawed, and centrifuged (5000 ´ *g*, 10 min, 4°C). The supernatant was used to confirm the presence of CHIKV by RT-PCR. 

The RT-PCR products from the CHIKV viral isolates (n = 7) were purified and sequenced by an automated Sanger sequencing. Contigs were generated using the CAP3 sequence assembly program, Pôle Rhône-Alpes de Bioinformatique (PRABI), following the assembly of both positive-sense and negative-sense strand DNA reads.

The phylogenetic analysis of the seven isolated strains was performed using the multiple sequence alignment (MSA) with 29 previously genotyped CHIKV sequences using Clustal Omega, EBI. The alignment was edited to remove gaps and non-paired extremities using the MEGA X software. A phylogenetic tree was generated using an IQ-Tree based on the inference of the Bayesian information criterion (BIC) using the K2P+G4 nucleotide substitution model and approximate likelihood-ratio test with 1000 pseudo-replicates. The online tool Interactive Tree Of Life (iTOL)^7^ was used to visualize and annotate the tree.

To analyze the phylogenetic relationship between all complete genome CHIKV strains available on GenBank, the Virus Pathogen Database and Analysis Resource (ViPR) was used to filter 662 GenBank sequences. These sequences were subjected to an MSA using the MAFFT, CBRC. The alignment was edited on the MEGA X software and imported to PhyML 3.0 (ATGC) to obtain a maximum likelihood (ML) tree based on the Akaike Information Criterion (AIC) inference, GTR+G+I as the substitution model, and neighbor joining (NJ) tree as the starting tree under the branch Support of SH-aLRT^8^, which is a robust statistic method to analyze big data. The generated tree was edited using iTOL.

As a result, we found that from the 605 patient samples obtained from 2014 to 2018 from Salvador and the nearest counties in Bahia with clinical records of an acute CHIKV-like symptoms, 167 were positive and 445 were negative for CHIKV in the RT-PCR analysis. 

The highest number of positive samples (38.63%) was from 2014 (Table 1) when one of the two CHIKV outbreaks was reported in Brazil. This case was in Feira de Santana, 100 km from Salvador. In 2016 (Table 1), a city in Salvador reported a large of number of positive samples (64%). According to the epidemiological reports of the Brazilian health authorities, the occurrence of CHIKV cases increased in the first half of the year^9^. However, our results show a different pattern, as CHIKV cases were mainly reported between June and November during the four consecutive years of this study. It has been suggested that the competence of *A. aegypti* or *A. Albopictus* mosquito vectors may vary at different times of the year for each location. This phenomenon may be associated with the regional dynamics such as climate and environmental conditions that may favor the proliferation, propagation, and even displacement of some specific Aedes species^10^.

**TABLE 1:** Screening of the CHIKV-positive serum samples from Bahia, Brazil, by RT-PCR.

Year	Number of samples	CHIKV-positive samples		County
		n	%	(Number CHIKV- Positive/Total)
2014	88	38	41,97	Feira de Santana (34/81)
				Salvador (4/7)
2015	287	54	19,15	Salvador (52/269)
				Camaçari (2/13)
2016	106	68	45,28	Salvador (68/106)
2017	95	4	4,21	Salvador (4/93)
2018	29	3	10,34	Coração de Maria (3/29)
Total	605	167	100	

Viral isolation from the positive samples yielded 20 viral isolates. Seven of these isolates were randomly selected, sequenced by an E2 fragment amplification, and deposited into the GenBank (Accession Numbers: MK474461, MK474462, MK474463, MK474464, MK474465, MK474466, and MK474467). The construction of an ML phylogenetic tree with the seven isolated strains and the pre-genotyped sequences of CHIKV revealed their clustering into four genotypes. The strains isolated in Bahia belong to the ECSA lineage with a high bootstrap reliability value (Figure 1A). The ML phylogeny of the circulating CHIKV-ECSA strains from four different states of Brazil, namely, Bahia, Pernambuco, Alagoas (in the Northeast), and Roraima (in the North) suggests their relationship. The isolated strain from the outbreak in Coração de Maria in 2018 (MK474465) shared a high homology with other isolated strains from Bahia. Moreover, the strains isolated in 2014 and 2017 from Boa Vista, Roraima, were related to those from the Northeast of Brazil (Figure 1B). 

**FIGURE 1:**
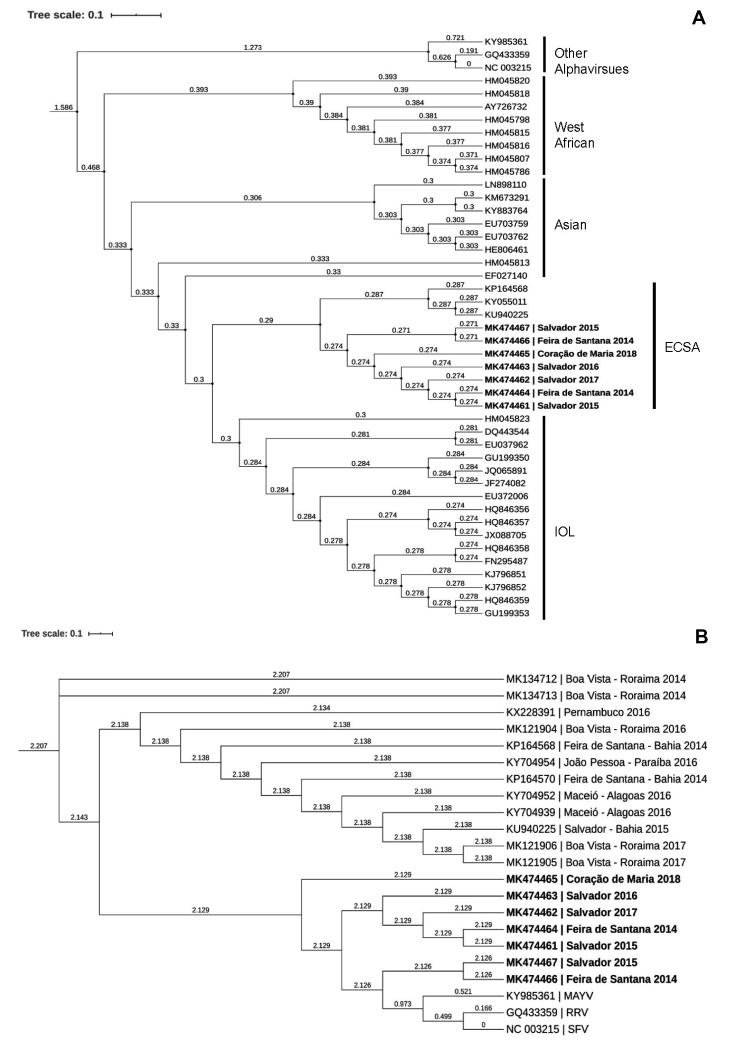
Phylogenetic genotyping of the isolated strains. Phylogenetic ML trees were constructed using the BIC and the K2P+G4 substitution model. The numbers on the left of the nodes indicate the branch lengths displayed as age, as inferred by the evolutive clock. (A) Phylogenetic genotype clusters of the isolated strains based on reference sequences. (B) Phylogenetic construction to evaluate the relationship between the ECSA strains isolated in Brazil between 2014 and 2018.

For a better understanding of the non-evident phylogenetic segregation between ECSA and IOL, we built a more robust ML tree using the complete sequences of all available CHIKV genomes in GenBank. As a result, we observed no phylogenetic differences between the ECSA and IOL strains, supporting that the complete sequence analysis did not indicated to diverge both lineages (Figure 2). These findings also show the predominance of the deposited complete sequences of AL (58.98%), followed by ECSA/IOL (42.74%) and a small proportion of the West African (2.26%) lineages.

**FIGURE 2:**
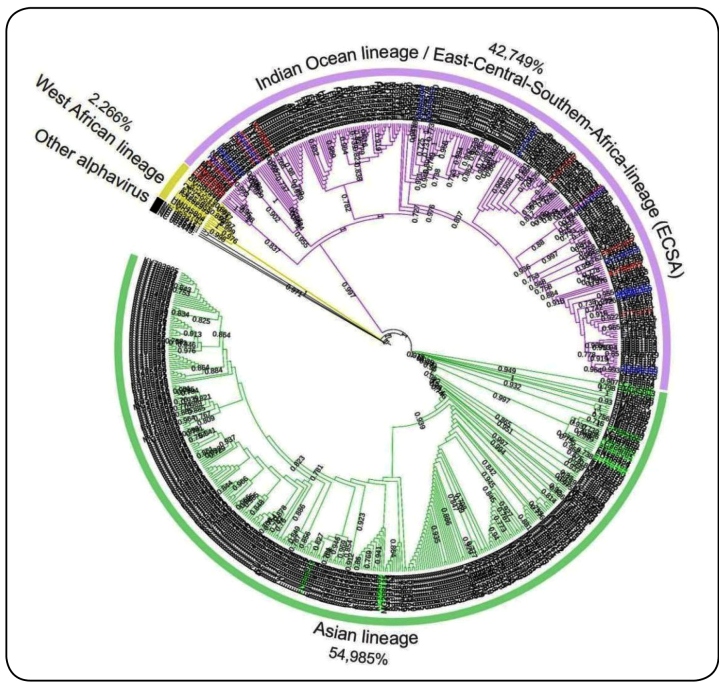
Phylogenetic genotyping of the available complete genome sequences of CHIKV. Phylogenetic ML trees were constructed using the SH-aLRT model to cluster different genotypes of CHIKV using all the complete genome sequences available on GenBank. Only the metadata values above 70% were considered and displayed to the left of the nodes. Colors in the taxon labels highlight the clustering of the different genotypes: AL (Green), IOL (Blue), ECSA (Red), WA (Yellow). The purple branch highlights the common cluster for both ECSA and IOL strains. The percentage on the genotype label refers to the frequency of strains available on GenBank in each group.

The molecular surveillance performed in this work provides valuable epidemiological information on the circulating strains of CHIKV. We could screen and isolate CHIKV from the human sera collected during the outbreaks in Feira de Santana in 2014^2^ and Salvador and Camaçari in 2015 and those obtained post-outbreaks until 2018^11^ ^,^ ^12^. We evaluated the frequency of positive cases via RT-PCR and revealed the correlation between the high frequency of positive cases in Feira de Santana in 2014 and the first outbreak of ECSA lineage in Brazil in the same year^2^. The decline in the frequency of positive cases after 2014, as reported in Salvador and Camaçari between 2015 and 2017, may be related to the improved government policy for vector control after the first outbreak. In 2015, Bahia had the first ZIKV outbreak of Brazil that may justify the increase in the number of samples owing to the uncertainty of the outbreak etiology, which was later confirmed as ZIKV^3^ ^,^ ^12^. In 2014 and 2015, a higher proportion of CHIKV-positive cases was detected in the Northeast states (39,851 reported cases). These included 83.3% of the recorded grievances, and 14,033 of these cases (29.3%) were confirmed^9^. This higher prevalence was associated with the higher number of reported cases in the state of Bahia, with the largest proportions in Feira de Santana (70.1%), Serrinha (74.2%), and Salvador (84.7%). 

The molecular characterization of the isolated strains from Bahia suggests the predominance of the ECSA lineage. This result correlates with the other reports on the predominance of the ECSA lineage in Feira de Santana that followed the first report on the entry of the ECSA lineage in the northeast of Brazil^2^. We failed to detect any AL in the samples from Bahia, suggesting that the CHIKV strains in the region predominantly belonged to the ECSA lineage. This observation is surprising, considering the rapid spread of AL from Oiapoque (Amapá state, north Brazil) since 2014 that led to the detection of CHIKV-AL in Boa Vista, Roraima^13^.

Analysis of the phylogeny of the available CHIKV complete genome sequences helped us to study the clustering of the CHIKV strains. Our analysis did not reveal the difference between the IOL and ECSA genotypes, supporting the idea that IOL was a part of the ECSA genotype. IOL was first acknowledged as a genotype separate from ECSA during an outbreak on the island of La Reunion in 2005-2006^14^. However, studies on the phylogenetic relationship between the CHIKV-IOL and CHIKV-ECSA lineages indicated that the strains isolated from the outbreaks in the Indian Ocean (2005-2006) were probably all derived from the ECSA strain^15^. In addition, the characteristic genomic microevolution of IOL, which highlights punctual amino acid changes in both structural and non-structural polyproteins, has been associated with the acquisition of a higher neurovirulence^15^. However, these punctual mutations were not enough to distinguish IOL from ECSA in the complete genomic phylogenetic analysis. 

To summarize our findings, we anticipate that the reported surveillance of CHIKV-positive cases from 2014 to 2018 will provide an overall vision of the viral epidemiology in the Bahia state, wherein the ECSA lineage remains predominant. Moreover, the amplification and sequencing of the short-nucleotide amplicon from the partial E2 gene of CHIKV may be applied as the phylogenetic inference method without next-generation sequencing. 
